# Antioxidant and Oxidative Stress: A Mutual Interplay in Age-Related Diseases

**DOI:** 10.3389/fphar.2018.01162

**Published:** 2018-10-16

**Authors:** Bee Ling Tan, Mohd Esa Norhaizan, Winnie-Pui-Pui Liew, Heshu Sulaiman Rahman

**Affiliations:** ^1^Department of Nutrition and Dietetics, Faculty of Medicine and Health Sciences, Universiti Putra Malaysia, Selangor, Malaysia; ^2^Laboratory of Molecular Biomedicine, Institute of Bioscience, Universiti Putra Malaysia, Selangor, Malaysia; ^3^Research Centre of Excellent, Nutrition and Non-Communicable Diseases (NNCD), Faculty of Medicine and Health Sciences, Universiti Putra Malaysia, Selangor, Malaysia; ^4^College of Veterinary Medicine, University of Sulaimani, Sulaimani, Iraq

**Keywords:** age-related diseases, healthy longevity, inflammation, oxidative stress, oxidative damage

## Abstract

Aging is the progressive loss of organ and tissue function over time. Growing older is positively linked to cognitive and biological degeneration such as physical frailty, psychological impairment, and cognitive decline. Oxidative stress is considered as an imbalance between pro- and antioxidant species, which results in molecular and cellular damage. Oxidative stress plays a crucial role in the development of age-related diseases. Emerging research evidence has suggested that antioxidant can control the autoxidation by interrupting the propagation of free radicals or by inhibiting the formation of free radicals and subsequently reduce oxidative stress, improve immune function, and increase healthy longevity. Indeed, oxidation damage is highly dependent on the inherited or acquired defects in enzymes involved in the redox-mediated signaling pathways. Therefore, the role of molecules with antioxidant activity that promote healthy aging and counteract oxidative stress is worth to discuss further. Of particular interest in this article, we highlighted the molecular mechanisms of antioxidants involved in the prevention of age-related diseases. Taken together, a better understanding of the role of antioxidants involved in redox modulation of inflammation would provide a useful approach for potential interventions, and subsequently promoting healthy longevity.

## Introduction

The average life expectancy has increased rapidly over the past decades, with an average of around 71.4 years in 2015 worldwide (World Health Organization, 2018). In view of the demographics of the world population in between 2000 and 2050, the population over 60 years is expected to grow from 605 million to 2 billion people (World Health Organization, 2014). Although the increasing life expectancy reflects a positive human development, a new challenge is arising. In fact, growing older is positively linked to cognitive and biological degeneration such as physical frailty, psychological impairment, and cognitive decline (Jin et al., 2015).

Age-related diseases have become the greatest health threats in the twenty-first century. Aging is an intrinsic, universal, multifactorial, and progressive process characterized as degenerative in nature, accompanied by progressive loss of function and ultimately increased mortality rate (Dabhade and Kotwal, 2013; López-Otín et al., 2013; Shokolenko et al., 2014; Chang et al., 2017). Among the theories that explain the aging process, the free radical theory of aging is long-established (Harman, 1956). This theory speculates that aging is a consequence of the failure of several defensive mechanisms to respond to the reactive oxygen species (ROS)-induced damage, particularly at the mitochondria (Islam, 2017). Age-related diseases are related to structural changes in mitochondria, accompanied by the alterations of biophysical properties of the membrane including alteration in the electron transport chain complexes activities, decreased fluidity, and subsequently resulted in energy imbalance and mitochondrial failure. These perturbations impair cellular homeostasis and mitochondrial function and enhance vulnerability to oxidative stress (Eckmann et al., 2013; Chistiakov et al., 2014). Elderly people are susceptible to oxidative stress due to a decline in the efficiency of their endogenous antioxidant systems. Organs such as brain and heart, with high rates of oxygen consumption and limited respiration levels, are particularly vulnerable to this phenomenon, hence partially explaining the high prevalence of cardiovascular diseases (CVD) and neurological disorders in elderly (Corbi et al., 2008).

Oxidative stress plays a crucial role in the development of age-related diseases including arthritis, diabetes, dementia, cancer, atherosclerosis, vascular diseases, obesity, osteoporosis, and metabolic syndromes (Tan et al., 2015a; Liu et al., 2017). ROS are generated within the biological system to modulate the cellular activities such as cell survival, stressor responses, and inflammation (He and Zuo, 2015; Zuo et al., 2015). Elevation of ROS has been associated with the onset and progression of aging. Although ROS generation may not be an essential factor for aging (López-Otín et al., 2013), they are more likely to exacerbate age-related diseases progression via oxidative damage and interaction with mitochondria (Dias et al., 2013). Due to their reactivity, high concentrations of ROS can cause oxidative stress by disrupting the balance of antioxidant and prooxidant levels (Zuo et al., 2015). Emerging research evidence has suggested that natural compounds can reduce oxidative stress and improve immune function (Ricordi et al., 2015). Indeed, oxidation damage is highly dependent on the inherited or acquired defects in enzymes involved in the redox-mediated signaling pathways. Therefore, the role of molecules with antioxidant activity that promote healthy aging and counteract oxidative stress is worth to discuss further. Of particular interest in this article, we highlighted the molecular mechanisms of antioxidants involved in the prevention of age-related diseases. An in-depth understanding of the role of antioxidants involved in redox modulation of inflammation would provide a useful approach for potential interventions, and subsequently promoting healthy longevity.

## Redox imbalance in age-related diseases

In the last few decades, several models have been suggested to define the interconnection and the biological pathways of aging (Dice, 1993). The widely accepted theory is the “oxidative stress hypothesis” (Ghezzi et al., 2017) that advanced and modified the free radical theory of aging (Harman, 1956). Based on the oxidative stress hypothesis, oxidative damage is not solely triggered by the unrestricted ROS production, but it also caused by other oxidants, such as reactive lipid species and reactive nitrogen species (RNS). The hypothesis of oxidative stress highlights the crucial role of antioxidant defenses as an important component of the overall redox balance of the organism. However, several studies demonstrated that avoiding oxidative stress damage does not increase longevity (Buffenstein et al., 2008; Pérez et al., 2009a,b).

Oxidative stress is considered as an imbalance between pro- and antioxidant species, which results in molecular and cellular damage (Conti et al., 2016). Mitochondria are major organelles that are accountable for generation of energy through oxidative phosphorylation to generate adenosine triphosphate (ATP), a molecule which is crucial for cellular actions (Weinberg et al., 2015). The electron transport chain consumes up to 90% of total oxygen (O_2_) taken up by the cells (Wallace, 2013). During this process, ROS are generated as by-products for the partial four-electron reduction of O_2_ to produce water molecule, which is the last electron acceptor in the ATP generation process (Ambrosio et al., 1993). Nearly 0.1–0.5% of inhaled O_2_ is converted to superoxide (${\text{O}}_{2}^{-}$) during the normal physiological states (Servais et al., 2009). In the normal healthy state, the generation and oxidation of ROS occur in a controlled manner. By contrast, the ROS production is increased under high-stress conditions or under disease states. The ROS generated from aerobic respiration caused a cumulative oxidative damage in macromolecules, including lipids, DNA, and proteins, which subsequently lead to cells death (Scheibye-Knudsen et al., 2015), and affect the healthspan of numerous principal organ systems (Dai et al., 2014).

An alteration of the redox status and the dysregulation of the immune system during aging may lead to the elevation of systemic inflammatory status. Both of these processes caused the activation of inflammatory mediators via oxidative stress-induced redox imbalance. The age-related redox imbalance is more likely triggered by the net effect of low antioxidative defense systems and incessantly produce of reactive species, including superoxide (${\text{O}}_{2}^{-}$), hydroxyl radical (•OH), peroxynitrite (ONOO^−^), hydrogen peroxide (H_2_O_2_), reactive lipid aldehydes, and reactive nitric oxide (NO) (Chung et al., 2009; Lennicke et al., 2015). Unresolved chronic inflammation during aging may serve as a pathophysiologic association which converts normal functional changes to the age-related degenerative diseases (Viola and Soehnlein, 2015). Oxidative stress is reinforced by several reactive species, including H_2_O_2_, singlet oxygen, other radicals, and non-radicals, which are consistently produced in the body due to the aerobic metabolism, and thereby potentially altering basic structural components such as proteins, lipids, and nucleic acids (Weidinger and Kozlov, 2015).

Template biosynthesis of polypeptide chains on ribosomes usually does not produce a functional protein. The newly developed polypeptide chain must undergo certain chemical modifications outside the ribosome. Thus, these modifications are most often accompanied by enzymes and take place after all the information supplied by the template RNA (mRNA) has been read, that is after mRNA translation. These additional processes are known as posttranslational modifications. There are four primary groups of protein functions which require posttranslational modification of amino acid residue side chains. The functional activity of several proteins requires the presence of certain prosthetic groups covalently bound to the polypeptide chain. These are usually involving complex organic molecules which take part in the protein activity for instance, the transformation of inactive apoproteins into enzymes. Another important group of modifications is protein tags, which provide intracellular localization of proteins such as marking the proteins for transport to the proteasome, where they will be proteolyzed and hydrolyzed. Additionally, some of the posttranslational modifications regulate biochemical processes by varying enzymatic activity (Knorre et al., 2009).

**Figure 1:**
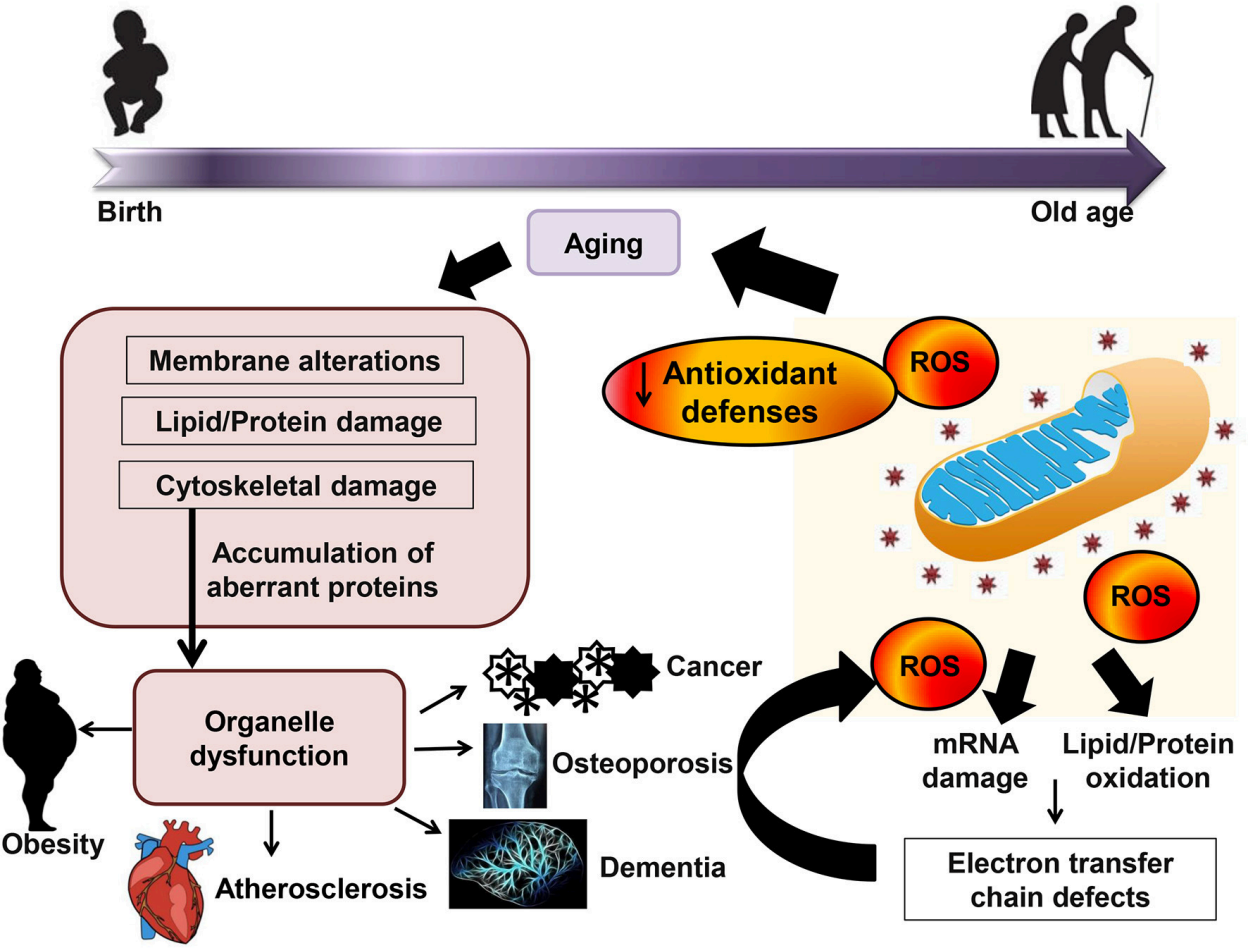
Effect of oxidative stress and the interaction of aging and age-related diseases. Accumulation of reactive oxygen species (ROS) leads to mRNA damage and lipid/protein oxidation and subsequently causes a decrease in mitochondrial function, and ultimately produces more oxidative stress. Mitochondrial function decline and oxidative stress response in aging may subsequently contribute to age-related diseases.

Naturally, the organism has several antioxidant defenses to protect against hostile oxidative environments, including classical antioxidant enzymes for example catalase, glutathione peroxidase, and superoxide dismutase as well as non-enzymatic ROS scavengers, such as β-carotene, vitamin C, vitamin E, and uric acid (Espinosa-Diez et al., 2015; Harris et al., 2015). Among all the antioxidant enzymes, glutathione peroxidase is the most powerful biological antioxidative reductant (Cross et al., 1977). Collectively, maintaining a healthy redox balance status is crucial for the physiological acid-base buffer system in the body for the optimal homeostatic cellular activities. Changing in redox balance would have a great impact on the transcriptional activities and cellular signaling pathways because most of the activation and reactions is dependent on the reduction/oxidation processes. Figure 1 shows the effect of oxidative stress and the interaction of aging and age-related diseases.

## Chronic inflammation and aging (inflammaging)

Inflammaging is a chronic, low-grade, and systemic inflammation in aging, which is occurred in the absence of overt infection (Franceschi and Campisi, 2014). Chronic inflammation is usually derived from the damaged cells or macromolecules due to an inadequate elimination or increased production. The ability of gut to sequester harmful microbes declines with age. Therefore, some of the harmful products that produced by the microbial constituents of the human body, such as gut microbiota, is capable to permeable into surrounding tissues (Biagi et al., 2011), and subsequently leading to chronic low-grade inflammation.

Senescence, a cellular response to stress and other damage (Franceschi and Campisi, 2014). Persistent senescent cells have been associated with aging or age-related diseases via secretion of proinflammatory cytokines that alter the tissue microenvironment or modify the function of normal cells (Baker et al., 2011). The study reported by Coppé et al. (2010) demonstrated that elimination of senescent cells in prematurely aged mice can prevent many age-related diseases. Increased inflammation may also derive from the stimulation of coagulation system. Coagulation is regarded as a part of the inflammation system. Aging promotes the hypercoagulable state and increased the risk of arterial and venous thrombosis in the elderly (Franceschi and Campisi, 2014). Additionally, aging also alters the immune system, which is subsequently leading to inflammaging. Adaptive immunity decreases with age; conversely, innate immunity demonstrated minute changes in mild hyperactivity (Santoro et al., 2018). The response of innate immunity might increase when adaptive immunosenescence progresses. These age-related changes could be due to the lifelong exposure to antigens and pathogens, as well as intrinsic changes in immune cells (Stephenson et al., 2018).

## Molecular inflammation involved during aging

Numerous age-related diseases undergo the inflammation process, which is a risk factor in or partly of disease development (DeBalsi et al., 2017). For instance, several age-related diseases including diabetes, dementia, metabolic syndrome, osteoporosis, cancer, arthritis, and cardiovascular diseases have been recognized as inflammatory disorders (Tan et al., 2015b; Abbas et al., 2017; Liu et al., 2017). The interaction between inflammation and oxidative stress is tightly associated with the prostaglandins (PGs) biosynthetic pathway that produces reactive species (Kawahara et al., 2015). PGs are lipid metabolites of arachidonic acid which have strong proinflammatory responses with pathogenic activities. For example, certain PG metabolites act as an active mediator of inflammation. While, some of the reactive species produced from PGs metabolism may exacerbate inflammation and induce tissue damage (Blaser et al., 2016). Cyclooxygenase (COX) is a predominant enzyme in the PG synthetic pathway, which produces prostaglandin H_2_ (PGH_2_) from arachidonic acid (Shehzad et al., 2015). Reactive species are generated during the conversion of prostaglandin G2 (PGG_2_) to prostaglandin H2 (PGH_2_) (Rashid, 2017). The production of reactive species via PG synthesis pathway contributes significantly to the overall reactive species pool in both pathological and normal states, especially during aging (Nita and Grzybowski, 2016).

Research evidence has suggested that the molecular inflammatory process plays a vitally important role during the aging process and age-related diseases (Davalli et al., 2016). COX-derived reactive species and transcriptional activity of interleukin-1beta (*IL-1*β), interleukin-6 (*IL-6*), tumor necrosis factor-α (*TNF-*α), cyclooxygenase-2 (*COX-2*), and inducible nitric oxide synthase (*iNOS*) are increased during aging (Michaud et al., 2013; Zhang and Jiang, 2015; Puzianowska-Kuznicka et al., 2016). Other pro-inflammatory proteins such as vascular cell adhesion molecule 1 (VCAM-1), P- and E-selectin, and intercellular adhesion molecule 1 (ICAM-1), are all enhanced during aging (Biswas, 2016).

The nuclear factor-kappa B (NF-κB) transcription factor has been identified as the key factor during inflammation which can be stimulated by oxidative stimuli. In fact, the stimulation of NF-κB-dependent genes is a principal culprit that is responsible for the systemic inflammatory process (Golia et al., 2014). Under high- stress circumstances, proinflammatory genes encode proinflammatory proteins, including chemokines, growth factors, and cytokines. NF-κB activity is mediated by numerous signaling pathways such as mitogen-activated protein kinases (MAPKs) and IκB kinase (IKK). The upregulation of IKK complexes phosphorylate the IκB subunits of NF-κB/IκB and subsequently activate the NF-κB (Jain et al., 2016). IKK activity is triggered during aging by NF-κB (Kim et al., 2002), which further modulates the p38 MAPK, extracellular signal-regulated kinase (ERK), and c-Jun N-terminal kinases (JNKs) pathways that modulate the NF-κB-dependent transcriptional activity during the inflammatory reaction. ROS production during the aging process has been associated with p38 MAPK, JNK, and ERK activities (Zhang et al., 2015). Nonetheless, uncontrolled input signal during aging may cause chronic proinflammatory conditions that are conducive to various chronic diseases (Fougère et al., 2017). Aging is also linked to the elevation of inflammatory cell (monocytes and neutrophil) counts and C-reactive protein (CRP) levels (Tang et al., 2017). High IL-6 plasma levels were shown to have a greater likelihood of mortality, morbidity, and disability in the elderly (Puzianowska-Kuznicka et al., 2016). Indeed, high plasma level of TNF-α is associated with a marked increase in CRP and IL-6, suggesting an interrelated stimulation of the entire inflammatory cascade (Xia et al., 2016).

In addition, compelling evidence suggests that DNA damage response (DDR) signaling is a predominant mechanism associated with the build-up of DNA damage, aging, and cell senescence (Malaquin et al., 2015). This study indicates the involvement of epigenetic modifications such as small, non-coding RNAs and microRNAs, which contributes to post-transcriptional regulation. These modifications have been hypothesized to play a crucial role in the diffusion of DNA damage response/senescence-associated secretory phenotype (DDR/SAPS) signaling to non-damaged surrounding cells during aging, suggesting that DDR/SASP signaling components may contribute to the development of novel therapeutic interventions against age-related diseases (Olivieri et al., 2015). Moreover, microRNAs may also be harnessed as an innovative tool to identify target senescent cells and to develop therapeutic interventions that can delay the proinflammatory programme stimulated in senescent endothelial cells (Prattichizzo et al., 2016).

## Accelerated-aging syndromes

Progerias or accelerated-aging syndromes are partially recapitulated normal aging (Burtner and Kennedy, 2010). Most of the accelerated-aging syndromes are induced by modification of nuclear envelope or by defects in DNA repair systems. Werner syndrome is the most common accelerated-aging syndrome derived from DNA repair defects, caused by the mutations of Werner syndrome ATP-dependent helicase (*WRN*), a gene coding for a protein implicated in telomere maintenance and homology-dependent recombination repair (Osorio et al., 2011). Another common accelerated-aging syndrome is Hutchinson-Gilford progeria syndrome (HGPS), caused by the defects in nuclear envelope proteins due to the mutations in the processing protease FACE1/ZMPSTE24 or genes encoding lamin A (Worman, 2012). Compared to HGPS, the onset of Werner syndrome is slightly slower, in which the pathology accompanies with Werner syndrome resembles a premature aging. Clinical pathology of Werner syndrome starting from 10 to 20 years of age including early graying, short stature, hair loss, and bilateral cataracts. The cellular phenotypes linked to the Werner syndrome demonstrate significant overlap with laminopathies. Further, cells in the absence of WRN have defects in DNA double-strand breaks, especially those bound with DNA replication fork arrest.

Interestingly, the generation of ROS is increased in HGPS fibroblasts (Viteri et al., 2010) and this phenomenon is similar to normal aged fibroblasts. High ROS level in HGPS cells could be attributed to the large DNA damage and subsequently resulting in an underlying defect in early senescence in HGPS cells (Huang et al., 2005; Gonzalez-Suarez et al., 2009). HGPS cells also show persistent markers of high basal DNA damage, such as nuclear ataxia telangiectasia mutated (ATM) foci (Liu Y. et al., 2006). The previous study showed that fibroblasts isolated from individuals with HGPS demonstrate lamin A has an ability to repair DNA lesions (Burtner and Kennedy, 2010).

Mutation in lamin A/C (*LMNA*) has been identified as the target gene for HGPS. Fibroblasts from patients with HGPS show increased levels of basal phosphorylated histone variant H2AX (γH2AX) and increased amounts of phosphorylated checkpoint kinase 1 (CHK1) and CHK2, compared with unaffected fibroblasts (Liu Y. et al., 2006). In addition, fibroblasts from individuals affected by HGPS, or from mice lacking ZmPSTe24, demonstrate a marked delay in the recruitment of p53 binding protein 1 (53BP1) to sites of DNA repair upon exposure to DSB-inducing irradiation (Liu et al., 2005). The delay in 53BP1 recruitment to DSBs in these cells and the accumulation of irreparable damage may be a potent physiological genotoxic stress in individuals with HGPS. Collectively, increased levels of DNA damage may have important consequences *in vivo*.

## Antioxidant and age-related diseases

Antioxidants control the autoxidation by interrupting the propagation of free radicals or by inhibiting the formation of free radicals via different mechanisms. These compounds help in scavenging the species that initiate the peroxidation, breaking the autoxidative chain reaction, quenching •${\text{O}}_{2}^{-}$, and preventing the formation of peroxides (Gaschler and Stockwell, 2017). The most effective antioxidants are those possessing the ability to interfere with the free radical chain reaction. They contain phenolic or aromatic rings which allow these antioxidants donate H• to the free radicals formed during oxidation. The radical intermediate is then stabilized by the resonance delocalization of the electron within the aromatic ring (Wojtunik-Kulesza et al., 2016).

Antioxidant plays a central role in the termination of oxidative chain reactions by removing the free radical intermediates (Gholamian-Dehkordi et al., 2017). Many studies indicate that cellular redox status is crucial for ROS-mediated signaling and mitochondrial function (Fang et al., 2018). Depletion of intracellular glutathione (GSH) markedly promotes mitochondrial ROS production and triggers mitochondrial membrane depolarization (Lohan et al., 2018). Stimulation of the Nrf2/ARE pathway is fundamental for the induction of antioxidant defense enzyme and the modulation of the intracellular GSH in response to stress (Liu et al., 2018a). Administration of *N*-acetylcysteine reverses GSH depletion and restores ARE-associated transcriptional activity to basal levels (Limón-Pacheco et al., 2007). Appropriate intracellular levels of ROS plays a crucial role in physiological redox signaling via activation and regulation of endogenous defenses by protecting cells from nitrosative, oxidative, and electrophilic stress (Moldogazieva et al., 2018). Indeed, supplementation with exogenous antioxidants depletes exercise-triggered improvements in insulin sensitivity and antioxidant gene expression (Ji et al., 2006), suggesting the importance of ROS induced endogenous antioxidant enzymes in restoring physiological redox balance. Additionally, overexpression of thioredoxin (Trx) has been demonstrated to inhibit the progression of insulin resistance in both type 1 and type 2 diabetes *in vivo* (Yamamoto et al., 2008). Recent findings suggest that a protective role of Nrf2 on oxidative stress in aging (de Oliveira et al., 2018). Depletion of Nrf2 activity has been identified to contribute to the development of age-related diseases (Cuadrado et al., 2018).

**Figure 2:**
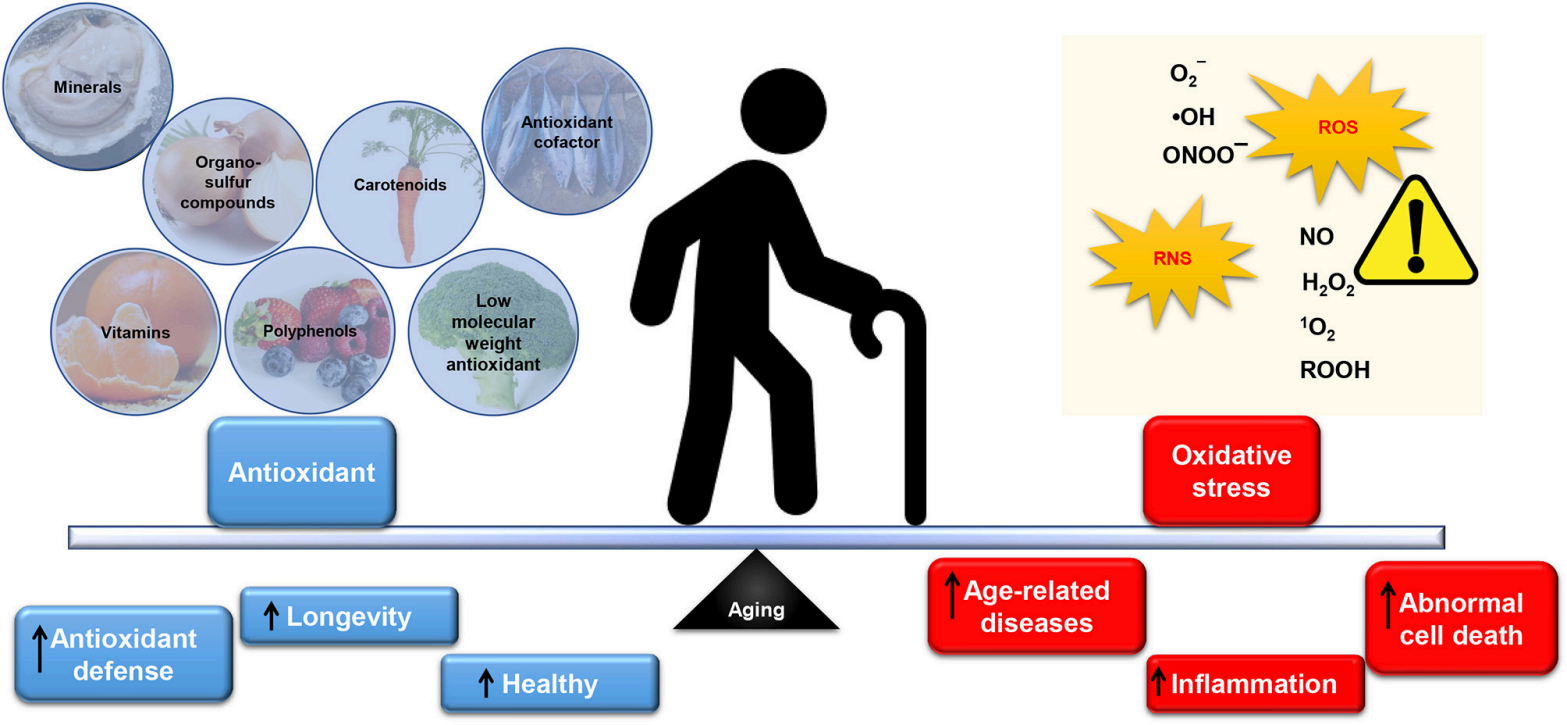
The balance of antioxidants and oxidative stress in aging. An inevitable by-product from aerobic respiration, reactive oxygen species (ROS) at the appropriate level is beneficial and essential for normal cell signaling and cellular immunity. Similarly, reactive nitrogen species (RNS) can be physiologically useful. In a normally functioning cell, antioxidants may adequately neutralize excess ROS/RNS. However, overproduction of reactive species, including superoxide (${\text{O}}_{2}^{-}$), hydroxyl radical (•OH), peroxynitrite (ONOO^−^), hydrogen peroxide (H_2_O_2_), hydroperoxides (ROOH), singlet oxygen (^1^O_2_), reactive lipid aldehydes, and reactive nitric oxide (NO) coupled with low level of antioxidants in the body may cause oxidative damage to the cellular constituents (protein, lipids, and DNA). This phenomenon is suffered by elderly and thereby promoted abnormal cell death, inflammation and subsequently contributes to age-related diseases. Substantial evidence has demonstrated the importance of antioxidants intake from dietary nutrients to replenish low level of antioxidants (especially endogenous antioxidant such as glutathione and coenzyme Q10) in the body. Antioxidant plays a pivotal role in scavenging ROS/RNS thus protecting the cells from oxidative damage. Administration of exogenous (minerals, organosulfur compounds, vitamins, carotenoids, polyphenols) and endogenous antioxidant (antioxidant cofactor such as coenzyme Q10; and low molecular weight antioxidant: glutathione) have shown to maintain the antioxidant defense and subsequently leads to healthy longevity.

Several studies as reported by Tan et al. (2018) have shown that oxidative stress and obesity-associated non-communicable diseases (NCDs) can be mediated by nutrient-rich in antioxidants. Indeed, a unique complex of bioactive constituents can provide protection against oxidative stress, which can cause in inflammation (Tan et al., 2015a,b; Tan and Norhaizan, 2017). In support of this, numerous epidemiological studies including European paradox study (Bellizzi et al., 1994), WHO/MONICA study (Gey and Puska, 1989), NHS study (Stampfer et al., 1993), and Harvard HPSF (Rimm et al., 1993) have shown that antioxidant was negatively associated with many NCDs including cardiovascular diseases. In this regard, the antioxidant capacity in natural products has drawn attention among scientists in academia and industry in the prevention of age-related diseases. Figure 2 summarizes the dietary intake of antioxidants in relation to oxidative stress in aging.

Mitochondria-targeted antioxidants have great potential against the damage caused by ROS generation. The ability of mitochondria-targeted antioxidants confers greater protection against oxidative damage has been attributed to their abilities to cross the phospholipid bilayer of mitochondria and thus eliminating ROS (Oyewole and Birch-Machin, 2015). In principle, a broad range of antioxidants could be targeted to mitochondria via conjugation of triphenylphosphonium (TPP) moiety (Smith and Murphy, 2011). In particular, ubiquinol (MitoQ) is the best-characterized antioxidant targeted to mitochondria by conjugation to the TPP cation (Smith and Murphy, 2011). The role of MitoQ will be described in the ubiquinone section.

## Role of antioxidants in the prevention of age-related diseases

### Low molecular weight antioxidant

Low molecular weight is defined as small molecule biological compound (< 900 daltons) which regulates body physiological process (Veber et al., 2002; Macielag, 2012). Low molecular weight antioxidants such as minerals, vitamins, carotenoids, cofactors, glutathione, and polyphenols are crucial for antioxidative defense mechanisms of cells and organisms (Grune et al., 2004). Ascorbic acid (vitamin C) and tocopherol (vitamin E) are the most important low molecular weight antioxidants that cannot be synthesized by a human (Podda and Grundmann-Kollmann, 2001). There are several molecules that are synthesized in the human body and possess an antioxidant effect including glutathione, lipoic acid, uric acid, taurine, keto acids, melatonin, coenzyme Q, and melanins. Among these antioxidants, glutathione is one of the major cellular antioxidant (Sifuentes-Franco et al., 2017).

### Glutathione

Glutathione is a pivotal antioxidant present in the microorganisms, plants, and animals. Glutathione prevents the cell damage induced by ROS including lipid peroxides, peroxides, free radicals, and heavy metals (Pisoschi and Pop, 2015). Glutathione can scavenge ROS via non-enzymatic and enzymatic reactions. The non-enzymatic antioxidant activity is contributed by the free thiol group of glutathione (Winterbourn, 2016). Additionally, glutathione also detoxifies oxidants and electrophiles via enzymatic reactions which involve glutathione reductase, glutathione peroxidase, and glutathione-S transferase (Farhat et al., 2018). Glutathione plays a crucial role in regulating redox state of the cell, specifically via modulation of the proper tertiary structure of proteins through thiol-disulfide exchange concomitantly with glutaredoxin and protein disulfide isomerases (Ye et al., 2017). Besides antioxidant properties, glutathione also involves hormones metabolisms such as estrogens, leukotrienes, and prostaglandins and signal transduction for transcription (Rotar et al., 2014). Alteration of glutathione concentration has been linked to adverse health impacts such as dysregulation of cell proliferation, transcription of detoxification enzymes, and apoptosis (Aquilano et al., 2014).

**Figure 3:**
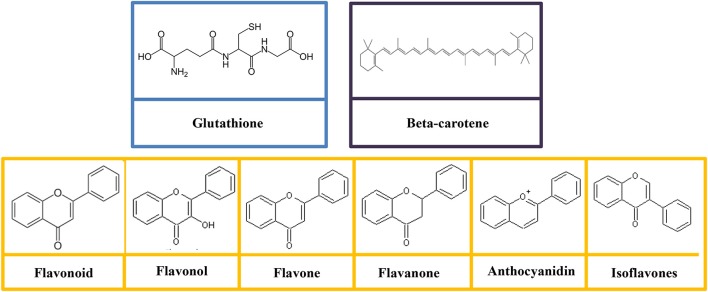
Molecular structures of glutathione, polyphenols (flavonoid, flavonol, flavone, flavanone, anthocyanidin, and isoflavones), and beta-carotene.

Glutathione is categorized as a non-essential nutrient for humans, as it can be synthesized in the body from the amino acids such as L-glutamic acid, L-cysteine, and glycine (Lu S. et al., 2016). The structure of glutathione consists of gamma peptide bond linked to a tripeptide between the amine group of cysteine and carboxyl group of the glutamate side-chain (Figure 3). The carboxyl group of cysteine is linked to a glycine by peptide bond (Gu et al., 2012). The sulfhydryl group of cysteine serves as a proton donor and allows the glutathione to act as an antioxidant (Gümüşay et al., 2015). Sulfur is the second chalcogen after oxygen in the periodic table with a vacant 3d orbital. The sulfur atom in the sulfhydryl functional group is in its low oxidation state (García-Santamarina et al., 2014), and thereby sulfhydryl is strongly susceptible to oxidation even without the presence of the enzyme.

Homeostasis of intracellular glutathione is not solely regulated by *de novo* synthesis, but it also by several factors including cellular export, utilization, and recycling (Lu S. C. et al., 2016). This redox cycle is recognized as the glutathione cycle which comprises of glutathione, together with other redox-related enzymes acts as the first defense against overproduction of harmful ROS in addition to repairing ROS-induced damage (Schieber and Chandel, 2014). There are three groups of enzymes involve in the glutathione cycle, namely glutathione reductase, glutathione oxidase, and glutathione peroxidase (Reczek and Chandel, 2015). By serving as an electron donor, glutathione reduces disulfide bonds formed between proteins and cytoplasm to cysteines. In this process, two molecules of glutathione are converted to an oxidized form, either glutathione oxidase or glutathione peroxidase. Once oxidized, glutathione reductase is capable to regenerate glutathione from glutathione disulfide via NADPH-dependent process (Reczek and Chandel, 2015). Further, glutathione tends to react with cysteine residues in proteins through the formation of mixed disulfides. Yet, these unstable molecules can easily be reverted to a normal state by glutathione S-transferase (Carvalho et al., 2016). Alternatively, the cells export glutathione disulfide to the extracellular medium to restore redox imbalance (Heidari et al., 2016). Indeed, the *de novo* synthesis of glutathione is essential for adaptive response toward oxidative stress. Many toxic by-products produced from normal cellular metabolism processes can be detoxified by glutathione. A toxic compound, methylglyoxal, generated from the glycolytic process is implicated in ROS production (Chan et al., 2016). Glutathione detoxifies methylglyoxal by serving as a cofactor of enzyme glyoxalases (Nahar et al., 2015). Besides scavenging ROS, glutathione is also involved in protecting against reactive nitrogen species-mediated damage (Cassia et al., 2018). NO reacts with the cysteine of glutathione to form S-nitroglutathione (GSNO). GSNO reductase converts GSNO to glutathione disulphide, and subsequently reduces to glutathione by glutathione reductase (Cassia et al., 2018).

The modulation of glutathione metabolism is a useful adjuvant therapy for many diseases such as cardiovascular diseases, diabetes, and brain disorders. A study analyzed of 134 cardiovascular disease cases involving 435 individuals revealed that glutathione can affect the risk of cardiovascular diseases (Shimizu et al., 2004). The data showed that the total plasmic glutathione content is lower in cardiovascular diseases patients (cerebral infarction and cerebral hemorrhage) compared to healthy subjects (Shimizu et al., 2004). Further, glutathione peroxidase was found to be inversely correlated with cardiovascular disease risk (Espinola-Klein et al., 2007). Although research has demonstrated a negative association between glutathione peroxidase and cardiovascular disease risk, not all data demonstrated such a link. Mills et al. (2000) did not identify an association of glutathione level and atherosclerosis.

Maintaining a normal level of glutathione is important for diabetic patients. Diabetes induces an alteration in glutathione peroxidase and glutathione reductase activity (González de Vega et al., 2016). Intake of glutathione in patients with type 2 diabetes mellitus increase the platelet constitutive nitric oxide synthase activity and reduce plasminogen activator inhibitor-1 (PAI-1; Martina et al., 2001). PAI-1 is an inhibitor of fibrinolysis which increases the risk of thrombosis (Tofler et al., 2016). This study indicates that glutathione may play a vital role in the pathophysiology of diabetes. Further, nephropathy is a life-threatening complication suffered by both Type 1 and Type 2 diabetes patients (Hadjadj et al., 2016). Diabetic nephropathy is often linked to low renal glutathione levels. Dietary supplementation with glutathione has been demonstrated to protect against pathologies associated with diabetic nephropathy (Lash, 2015).

In the brain, glutathione is only presented in millimolar concentrations, and thus makes this organ prone to oxidative damage compared to other tissues in the body (Settineri et al., 2018). A disruption in glutathione homeostasis could induce oxidative stress and lead to neurodegenerative diseases including Parkinson's disease (Mischley et al., 2013), Alzheimer's disease (Braidy et al., 2015), and dementia (Duffy et al., 2015) which impaired motor and cognitive functions. Parkinson's disease is a dopamine deficiency condition resulted from the destruction of dopaminergic neurons in the midbrain region (Zucca et al., 2017). ROS is generated during dopamine normal metabolism (Guo et al., 2018). The decline of glutathione levels in Alzheimer's patient was associated with downregulation of glutathione homeostasis (Braidy et al., 2015). Parkinson's patients suffer depletion of glutathione levels coupled with an increase of ROS within the midbrain (Mischley et al., 2015). Removal of ROS by glutathione can facilitate the regulation of redox potential of the midbrain. In a clinical condition, patients with Parkinson's disease were improved following supplementation of reduced glutathione (Mischley et al., 2013, 2015).

As opposed to the role of the other antioxidants, glutathione has a complex function in the cancer cells. The level of glutathione was elevated in several human cancer cells such as colon, bone marrow, breast, and lung cancers. In colon cancer cells, the mRNA and protein expressions of glutathione and enzyme involved in the metabolism of glutathione were significantly higher as compared to the normal colonic cell line (Kim et al., 2015). In support of this, 60% of colon cancer patients expressed high levels of glutathione particularly in tumor tissue (Kim et al., 2015). Similarly, the study reported by Harris et al. (2015) has also revealed that glutathione is recruited especially during cancer initiation. Interestingly, glutathione synthesis is only effective during the early stage of cancer, it is not observed in the established tumor (Nguyen et al., 2016). Due to glutathione antioxidant activity, there has been a tremendous interest in the study of glutathione and its related compounds in diseases suffered by elderly. The vital role played by glutathione is nonetheless worth study in-depth in order to understand the pathophysiology pathway underlying in age-related diseases.

### Polyphenol

Polyphenols (also known as polyhydroxyphenols) are characterized by the multiples of phenol structural units (Nascimento-Souza et al., 2018). The numbers and characteristics of these phenol structures contribute to the unique features of polyphenol compound in term of chemical, physical, and biological (Scalbert and Williamson, 2000). In brief, polyphenols are secondary metabolites (Kabera et al., 2014) produced by plants, which is widely found in fruits and vegetables are thought to protect against ultraviolet radiation (Zbikowska et al., 2016) and pathogens invasion (Nagpala et al., 2016). Polyphenols also affect the flavor, color, and odor which contribute to the sensory perception of the food (Ju et al., 2017). Of all polyphenolic compounds, the flavonoid is the most common polyphenol classes (Nascimento-Souza et al., 2018).

Flavonoids are comprised of the most studied group of polyphenol. The basic structure of flavonoid is a diphenylpropane skeleton, which composed of two benzene rings (rings A and B) connected by three-carbon chains that form a closed pyran ring (heterocyclic ring containing oxygen, ring C; Das et al., 2017). The structure of flavonoids is denoted as C6-C3-C6 (Das et al., 2017). Generally, B ring is bonded to position 2 of C ring, but some of them are attached at position 3/4. Flavonoids are subdivided into different subgroups (flavonols, flavones, flavanonols, flavanones, catechins, anthocyanins, and chalcones; Figure 3) based on the carbon and the degree of unsaturation (Gonzales et al., 2015). The physiological function of the flavonoids is depends on the structural characteristics as well as the pattern of glycosylation and hydroxylation of the three rings (Gonzales et al., 2015).

There are around 6,000 flavonoids that form the colorful pigments of herbs, fruits, vegetables, and medicinal plants. Flavonoids are known for its broad spectrum of health-promoting effects on human and animal (Panche et al., 2016). The antioxidative, antimutagenic, anti-inflammatory, and anticarcinogenic properties coupled with their abilities to regulate key cellular enzyme function have drawn attention from the pharmaceutical industry, which attempts to design the prevention and treatment of certain diseases (Panche et al., 2016). The antioxidant activities of flavonoids include (1) scavenging ROS (Shokoohinia et al., 2015); (2) suppressing generation of ROS by inhibition of enzymes (Nile et al., 2016) and chelating trace elements (Catapano et al., 2017); and (3) upregulating antioxidant defenses. The low redox potential of flavonoids enables the reduction of highly oxidized free radicals such as superoxide, alkoxyl, hydroxyl, and peroxyl radicals by proton donation (Kovacic and Somanathan, 2011). Flavonoids inhibit the enzymes such as xanthine oxidase (Nile et al., 2016) and protein kinase C (Maurya and Vinayak, 2015) which are responsible for the generation of superoxide anion. Flavonoids have also been reported to inhibit other ROS generating enzymes including COX, microsomal monooxygenase, lipoxygenase, mitochondrial succinoxidase, and NADH oxidase (Pietta, 2000). Therefore, the ability of flavonoids in chelating trace metals plays an important role in the oxygen metabolism (Catapano et al., 2017).

Flavonoids have beneficial biochemical and antioxidant effects in relation to several oxidative stress-induced diseases in elderly for instance cancer (Chien et al., 2015), diabetic (Ding et al., 2014), cardiovascular diseases (Roohbakhsh et al., 2015), Alzheimer's disease (Swinton et al., 2018), and dementia (Swinton et al., 2018). Several flavonoids such as naringin (Ahmad et al., 2015), apigenin (Erdogan et al., 2016), and isorhamnetin (Manu et al., 2015) have been demonstrated to reduce the inflammatory mediators production via the blockade of NF-κB pathway. Flavonoids were found to negatively correlate with several types of cancer in human based on numerous studies. In woman aged 75 years old and above, high total flavonoids intake reduced the risk of cancer mortality compared to those with low total flavonoids consumption (Ivey et al., 2015). A decrease in breast cancer risk among postmenopausal women was found to be associated with flavonoids intake specifically, flavan-3-ols, flavones, flavonols, and lignans (Fink et al., 2006). While for colorectal cancer, He and Sun (2016) found that 2 flavonoid subclasses, namely procyanidins and isoflavones exert preventive effects toward the risk of colorectal cancer. However, there was limited evidence of the lower risk of colorectal cancer via flavonoid consumption (He and Sun, 2016; Grosso et al., 2017; Zamora-Ros et al., 2017).

Many studies demonstrated that a flavonoid-rich diet is related to a lower risk of cardiovascular disease. Flavonoids prevent cardiovascular disease via a few mechanisms such as antioxidant, anti-inflammatory, antiplatelet, and increasing high-density lipoprotein (HDL) level (Nunes et al., 2016). A study found that the intake of soy isoflavone reduces the risk of cardiovascular disease due to chronic inflammation. This favorable effect could be attributed to the downregulation of the TNF-α at the endothelial level (Nadadur et al., 2016). Studies have shown the ability of isoflavone to alleviate hypertension via modulation of vasodilation. Isoflavone improves brachial artery flow through interaction with the estrogen-response element of genes related to endothelial NO synthase (Ramdath et al., 2017). Compared to those who consume placebo, supplementation of isoflavone in postmenopausal women for 6 months improved endothelial vasodilation and lowered the cellular adhesion molecules such as E-selectin, ICAM-1, and vascular cell adhesion protein 1 (Colacurci et al., 2005). A recent study by Grosso et al. (2017) showed that intakes of dietary flavonoids (flavonols, flavones, flavanones, anthocyanidins, and proanthocyanidins) are associated with decreased risk of cardiovascular disease mortality. These data suggest that dietary flavonoids as natural cardiovascular protectors.

Diabetes mellitus is suffered by elderly and can lead to severe complication such as diabetic peripheral neuropathy. A study reported by Ganugapati et al. (2011) showed that green tea flavonoids and epicatechin activate the insulin receptor and reduce the harmful effects of diabetes. Grape seed proanthocyanidin alleviates type 2 diabetes mellitus in the rat through ameliorating of hyperglycemia and increases Ca^2+^-ATPase activity in sciatic nerve (Ding et al., 2014). Quercetin is another bioflavonoid available in red wine and many plants. Intake of quercetin was shown a neuroprotective effect in the diabetic rats against high glucose-induced injury on the glia and myenteric neurons at the cecum. The neuroprotective effects could be attributed to NF-κB inhibition and nuclear factor E2-related factor 2/heme oxygenase*-*1 (Nrf-2/HO-1) activation (Sandireddy et al., 2016). In another study, Kwak et al. (2017) revealed that baicalein, a flavonoid found in traditional Chinese herbal medicine can inhibit the oxidative-nitrosative stress and p38 MAPK activation and subsequently lead to alleviation of diabetic peripheral neuropathy.

Many studies revealed that flavonoids from cocoa (Swinton et al., 2018), green tea (Swinton et al., 2018), and citrus fruit (Braidy et al., 2017) exert beneficial effects to the brain. Emerging evidence has suggested that flavonoids protect against neural injuries and degeneration in Alzheimer's disease and dementia. In the brain, flavonoids act as a potent antioxidant, anti-inflammatory, anti-apoptotic and signaling pathways modulatory agents via interactions with the ERK and PI3-kinase/Akt signaling pathways (Jiang et al., 2016). Further, increased cerebral brain blood flow by flavonoids may also enhance cognition (Grassi et al., 2016). In addition, flavonoids have also been reported to slow down the development of Alzheimer's disease-like pathophysiology and related neurodegenerative disorders through disrupting amyloid β protein production, activating of α-secretase (ADAM10), and inhibiting of β-secretase (BACE-1) (Folch et al., 2018). Together, the evidence showed that flavonoids have outstanding potential to block the initiation and progression of age-related diseases and pathologies. High intake of flavonoids should be included in the dietary of elderly via supplementation or flavonoid-rich containing food. Table 1 summarizes some of the clinical trials of antioxidants in preventing age-related diseases and the failure of clinical trials involving antioxidants.

**Table 1:** Clinical studies conducted in several antioxidants and their effects in age-related diseases.

**Antioxidants**	**Age-related diseases**	**Findings**	**References**
Glutathione	Atherosclerosis	No effect	Mills et al., 2000
	Cardiovascular disease	Total plasmic glutathione content is lower in cardiovascular diseases patients (cerebral infarction and cerebral hemorrhage) compared to healthy subjects	Shimizu et al., 2004
		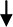 Cardiovascular disease risk	Espinola-Klein et al., 2007
	Type 2 diabetes mellitus	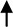 Platelet constitutive nitric oxide synthase activity and reduce plasminogen activator inhibitor-1 (PAI-1) Protect against pathologies associated with diabetic nephropathy	Martina et al., 2001; Lash, 2015
	Alzheimer's patient	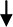 Glutathione levels in Alzheimer's patient were associated with downregulation of glutathione homeostasis	Braidy et al., 2015
	Parkinson's disease	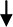 Free radicals involved in neurological complications	Mischley et al., 2015
	Cancer	60% of colon cancer patients expressed high levels of glutathione particularly in tumor tissue	Kim et al., 2015
Polyphenols	Cancer	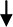 Risk of colorectal cancer	Grosso et al., 2017; Zamora-Ros et al., 2017
	Cardiovascular disease	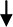 Risk of cardiovascular disease mortality	Grosso et al., 2017
		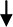 Vascular endothelial dysfunction and regulating lipid metabolism	Rasines-Perea and Teissedre, 2017
	Diabetes	Improve glucose control and insulin sensitivity	Vitale et al., 2018
Carotenoids	Alzheimer's disease and dementia	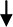 Risk of Alzheimer's disease mortality and dementia	Min and Min, 2014; Feart et al., 2015
	Rheumatoid	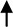 Mortality rate	Bjelakovic et al., 2012
	Cardiovascular disease	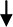 Risk in stroke, coronary artery disease, and cardiovascular disease	Leermakers et al., 2016; Valderas-Martinez et al., 2016
		Inversely correlated with oxidized LDL	Nakazato et al., 2014
		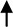 Mortality rate	Bjelakovic et al., 2012; Kishimoto et al., 2017
	Hypertension	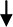 Baseline blood pressure	Perrone et al., 2016
	Age-related macular degeneration	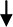 Risk in individuals who consume a carotenoid-rich diet	Eisenhauer et al., 2017
	Osteoporosis	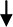 Bone density and fracture risk	Rao and Rao, 2015; Hayhoe et al., 2017
		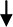 Hip fracture risk by 28%	Xu et al., 2017
	Cancer	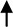 Lycopene intake can prevent prostate cancer	Zu et al., 2014
		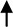 The incidence of lung cancer in the treatment group (30 mg of beta-carotene and 25,000 IU of retinol daily) in the smokers and workers in asbestos mines compared to the placebo group	Omenn et al., 1994
		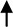 The incidence of lung cancer in male smokers (50–69 years) who received beta-carotene	Blumberg and Block, 1994
Zinc	Type 2 diabetes mellitus	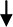 Plasma thiobarbituric acid reactive substances	Anderson et al., 2001
		Improved insulin sensitivity	Vashum et al., 2014
	Age-related macular degeneration	Delay the development of age-related macular degeneration and vision loss in individuals older than 55 years	Group, 2001; Gorusupudi et al., 2017
		Low intake of zinc was associated with age-related macular degeneration	Aoki et al., 2016
Ascorbic acid	Diabetes mellitus and coronary artery disease	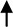 Forearm vasodilator response	Antoniades et al., 2004
	Cardiovascular disease	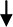 Cardiovascular disease	Wang et al., 2013
		Supplementation with a dosage >500 mg/d shows a better endothelial function	Ashor et al., 2014
		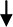 Endothelial dysfunction and improve lipid profile	Moser and Chun, 2016
	Age-related neurodegenerative diseases	Ascorbate shortage may contribute to the dysregulation of 5 hmC	Al-Mahdawi et al., 2014
	Cancer	Exert antitumor activity	Ma Y. et al., 2014; Yun et al., 2015
Tocopherols and tocotrienols	Cancer	No effect	Lonn et al., 2005
	Alzheimer's disease	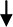 Lipid peroxidation by up to 60% compared with that of the control	Morris et al., 2005
		Positively associated with perceptual speed	Hensley et al., 2011
		Stimulate phosphoprotein phosphatase 2A (PP2A)	Voronkov et al., 2011
	Cardiovascular disease	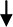 Cardiovascular mortality risk	Schwingshackl et al., 2017
		No effect	Lonn et al., 2005
	Myocardial infarction	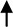 Chronic heart failure in patients with left ventricular dysfunction	Marchioli et al., 2006
	Vascular disease	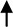 Risk of heart failure	Wannamethee et al., 2013
	Osteoporosis	Positive relationship between bone mineral density and α-tocopherol level in an elderly Chinese population	Shi et al., 2016
Ubiquinone	Parkinson's disease	Slow down the functional decline experienced by early-stage of Parkinson's disease patients	Shults et al., 2002
		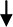 Cellular pathophysiological alterations linked to a mitochondrial dysfunction in Parkinson's disease patients	Cooper et al., 2012
		No effect	Snow et al., 2010
	Type 2 diabetes mellitus	Enhances nerve conduction parameters of diabetic polyneuropathy and ameliorates oxidative stress	Hernández-Ojeda et al., 2012
		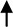 Nitric oxide production in patients received 200 mg CoQ10/day for 12 weeks	Watts et al., 2002
		Increases insulin sensitivity and improves beta cell function in diabetic patients	Raygan et al., 2016
		Improve vascular dysfunction and decrease the glycemic response	Mantle, 2017
	Coronary artery disease	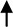 Antioxidant enzymes activities and 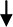 inflammation	Lee et al., 2013
	Congestive heart failure	Improve the quality of life in patients	Oleck and Ventura, 2016
		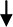 Risk of mortality	Lei and Liu, 2017
Sulfur compounds	Type 2 diabetes mellitus	Improve glucose control	Sobenin et al., 2008
		No hypoglycemic effects	Afkhami-Ardekani et al., 2006

### Carotenoids

Carotenoids are naturally occurring organic pigments that are produced in the plastids of plants and algae, several bacteria, and fungi (Alós et al., 2016). The only animals known to produce carotenoids are spider mite (*Tetranychus urticae*) and the red pea aphid (*Acyrthosiphon pisum*), which have acquired the ability to synthesize the carotenoids from fungi via gene transfer (Du et al., 2017). In general, carotenoids absorb wavelengths between 400 and 550 nanometers, thus the compounds appear in yellow, orange, or red color (Gauger et al., 2015).

Currently, there are more than 600 carotenoids that have been discovered to perform a range of functions (Paliwal et al., 2016). Carotenoids are divided into two classes, namely carotenes and xanthophylls based on their chemistry constitute (Yaroshevich et al., 2015). Hydrocarbon-only carotenoids (α-carotene, β-carotene, and lycopene) are known as carotenes (Figure 3); whereas oxygenated derivatives are called xanthophylls. On the other hands, oxygen substituents (lutein and zeaxanthin), keto/oxo groups (echinenone and canthaxanthin), epoxide groups (violaxanthin, antheraxanthin, and neoxanthin), and aldehyde groups (β-citraurin) are classified as complex xanthophylls (Berman et al., 2015).

Most carotenoids are tetraterpenoids, derive from 8 isoprene molecules and contain 40 carbon atoms (Harrison and Curley, 2016). All carotenoids have polyisoprenoid structure comprised of a long-conjugated chain adjacent toward the multiple double bonds with near symmetry on the central double bond. This basic acyclic structure can be altered by oxygen-rich functional groups (Gabriel et al., 2015). The electron-rich conjugated system of the polyene structure allows the carotenoids function as efficient radical scavengers by quenching the singlet oxygen and trapping peroxyl radicals (Nishino et al., 2016).

Carotenoids are not only exerted antioxidant properties, they are also facilitated in the modulation of cell cycle, apoptosis, and cell differentiation (Gloria et al., 2014), enhancement of immune system (Karadas et al., 2016), regulate of cell signaling pathways (Kim et al., 2016), promote growth factors (Diener and Rohrmann, 2016), and adhesion molecules (Llorente et al., 2017). Carotenoids are highly lipophilic molecules that reside intracellularly to shield the membrane from oxidative stress (Fiedor and Burda, 2014). Carotenoids are well-recognized as an eye-sight protecting agent. Such carotenoids are classified as pro-vitamin A which contains unsubstituted β-ionone ring (α-carotene, β-cryptoxanthin, β-carotene, and γ-carotene) and can be converted into retinal (Sandmann, 2015). Vitamin A deficiency affects immunity and subsequently leads to the damage of light-sensitive receptors (Gonçalves et al., 2016). Individual with vitamin A deficiency may acquire a permanent blindness known as xerophthalmia (West, 2015). Carotenoids such as lutein and zeaxanthin which is localized in the eye macula may protect against harmful blue and near-ultraviolet light (Ma et al., 2016). Age-related macular degeneration (AMD) is the main cause of blindness suffered by people aged 75 years and above in developed countries. AMD accounts for nearly 8.7% of all blindness worldwide (Wong et al., 2014). Research findings have predicted that the percentage of patients with AMD tends to double between 2010 and 2050 (Eisenhauer et al., 2017). Oxidative stress within the retina has been implicated in the pathogenesis of AMD. Compared to the other cells, non-proliferative postmitotic cells such as photoreceptors and retinal pigment epithelium cells are extremely sensitive to oxidative damage due to the absence of DNA damage detection systems (Blasiak et al., 2014). Further, the macular environment can also stimulate ROS generation. The macula is continuously exposed to high oxidative stress from the high partial pressure of choriocapillaris and oxidized polyunsaturated fatty acids (PUFAs) of the retinal outer segments (Schmidt-Erfurth, 2005). Compared to those who never or rarely consume carotenoids, individuals who consume a carotenoid-rich diet have a relatively low risk of age-related macular degeneration (Eisenhauer et al., 2017).

In addition to the oxidants scavenging ability, lutein also inhibits the activation of NF-κB which plays a significant role in the pathogenesis of various human diseases. NF-κB enters the nucleus, downregulates the inducible gene transcription and triggers the production of inflammatory markers such as iNOS, chemokines, and cytokines (Serasanambati and Chilakapati, 2016). The antioxidant and anti-inflammatory properties of lutein are not limited to only eyes but it also decreases the risk of cardiovascular diseases (Maria et al., 2015), coronary artery disease (Nakazato et al., 2014), and cancers (Rafi et al., 2015) in older people. Previous studies have demonstrated that intake of lutein was inversely correlated with oxidized LDL, suggesting that lutein may protect against the development of atherosclerosis (Kishimoto et al., 2017). Increased plasma lutein levels also decrease baseline blood pressure, which subsequently reduces the risk of hypertension (Perrone et al., 2016). The data from the previous study further demonstrated that lutein shields the myocardium from ischemia injury by reducing apoptosis and oxidative stress (Maria et al., 2015). A meta-analysis conducted by Leermakers et al. (2016) showed that high dietary intake of lutein is negatively linked to stroke and coronary heart disease. However, Bjelakovic et al. (2012) reported that β-carotene increases the mortality rate of cardiovascular disease and rheumatoid.

Lutein not only reduces cardiovascular disease but it also inhibits age-related cancers such as breast cancer via modulation of NrF2/ARE and NF-κB pathways (Chang et al., 2018). Another common carotenoid, lycopene is widely accepted as a potent antioxidant and reduces the risk of certain cancers such as lung (Aizawa et al., 2016), prostate (Graff et al., 2016), colon (Huang R. F. et al., 2015). Lycopene suppresses the progression of carcinogenesis via its anti-inflammatory actions (Carini et al., 2017). A follow-up study conducted from 1986 to 2010 involving 49,898 of males revealed that higher lycopene intake can prevent prostate cancer (Zu et al., 2014). The preventive role of lycopene toward cancer is more likely due to its antioxidant effect. Yet, the anticancer ability of lycopene is mediated through several mechanisms including modulation of cell cycle arrest, apoptosis, growth factor signaling, and phase II detoxifying enzymes (Aizawa et al., 2016). However, several studies demonstrated that smokers or workers in asbestos mines who received β-carotene or α-tocopherol alone is susceptible to lung cancer compared to the placebo group (Blumberg and Block, 1994; Omenn et al., 1994).

Bone loss in the elderly leads to osteoporosis. Studies in both human and animal models have suggested that carotenoids could reduce the risk of osteoporosis (Rao and Rao, 2015). Carotenoids have shown a positive impact on bone cells. For instance, β-carotene was significantly inhibited the bone marrow-derived macrophages viability (Wang et al., 2017). Beta-carotene decreased the receptor activator of nuclear factor kappa B ligand (RANKL)-induced osteoclastogenesis via inhibition of NF-κB pathway (Wang et al., 2017). Other carotenoids such as β-cryptoxanthin, α-carotene, lutein, and lycopene also facilitate the alleviation of bone loss. A meta-analysis involving 140,265 participants and 4,324 cases suggested that high dietary intake of total carotenoids reduced the hip fracture risk by 28% (Xu et al., 2017). Another study conducted by Hayhoe et al. (2017) also showed that bone density and fracture risk is inversely correlated with dietary intake of carotenoids.

Carotenoids have been demonstrated to prevent many degenerative diseases induced by an oxidative stress such as Alzheimer's disease and dementia (Mohammadzadeh Honarvar et al., 2017). The implication of carotenoids toward the pathophysiology of Alzheimer's disease and dementia has been extensively studied in both *in vitro* and *in vivo* models (Masisi et al., 2016). Carotenoids delay disease progression via multiple pathways such as suppress oxidative stress (Wang et al., 2018), promote Aβ peptide production (Lin et al., 2017), and inhibit pro-inflammatory cytokines (Hadad and Levy, 2017). Beta-carotene is an Alzheimer's disease antagonist due to its high binding energy toward Alzheimer's disease-related receptors (P53 kinase receptor and histone deacetylase; Krishnaraj et al., 2016). A marine carotenoid, fucoxanthin suppresses Aβ formation and destabilizes Aβ fibril (Xiang et al., 2017). A study reported by Ono and Yamada (2012) further revealed that both vitamin A and β-carotene can block the oligomerization of Aβ40 and Aβ42 during Aβ peptide formation. Another carotenoid, lycopene was shown to reduce the Aβ42-induced inflammatory cytokine such as IL-1β, NF-κB, transforming growth factor beta (TGF-β), and TNF-α in the brain (Sachdeva and Chopra, 2015). Data from the human studies revealed that higher plasma levels of lutein reduced the risk of Alzheimer's disease and dementia (Feart et al., 2015). High level of carotenoids (lutein, zeaxanthin, and lycopene) in serum has also been associated with a lower risk of Alzheimer's disease mortality (Min and Min, 2014).

The health-promoting values of carotenoids revealed the link between carotenoid-rich diets and age-related illnesses. The intake of raw tomato (*Solanum lycopersicum*) shields against cancers (esophagus, stomach, colon, and rectum) (Berman et al., 2015), cardiovascular diseases (Valderas-Martinez et al., 2016), as well as Alzheimer's disease (Oboh et al., 2015). Based on the evidence, dietary intakes of certain antioxidants such as carotenoids can reduce the risk of age-related diseases. The effects of multiple carotenoids in diet offer healthy aging in term of nutrition.

### Dietary minerals

Minerals are naturally occurring elements with universal structures and definite chemical formulas. Dietary minerals are the chemical substances required by all living organisms. Adequate intake of each dietary mineral is essential to maintain physical health. Minerals play a crucial role in bone formation, hormones synthesis, regulation of heartbeat and others (Morris-Naumann and Wark, 2015). Most of the minerals in human diet come from food and drinking water. Mineral supplements are made available in the market for those who did not meet the daily dietary intake of mineral (Schwalfenberg and Genuis, 2015).

Dietary minerals are categorized into two different groups, which are macrominerals and trace minerals. Macrominerals including phosphorus, calcium, sodium, magnesium, potassium, and chloride in which the body needs in larger amounts. By contrast, trace elements are dietary minerals that are required in minimal amounts for regular cellular function, such as copper, selenium, zinc, iodine, fluoride, and iron (Siddiqui et al., 2014). Most of these trace elements are the functional part of enzymes. Yet, intakes of a large amount of trace elements are noxious to both human and animals (Mikulewicz et al., 2017). For instance, trivalent chromium is responsible for glucose metabolism by acting as a cofactor for insulin action. However, massive inhalation of hexavalent chromium, a toxic industrial pollutant is carcinogenic to both animals and human. Chromium exposure has been associated with various cancers in lung, central nervous system, and gastrointestinal tract (Bhattacharya et al., 2016).

Minerals such as copper, magnesium, zinc, and selenium possess antioxidant properties. Zinc functions as an antioxidant in the body via regulation of glutathione metabolism (Stelmach et al., 2014), inhibition of nicotinamide adenine dinucleotide phosphate-oxidase (NADPH-oxidase) enzyme (Marreiro et al., 2017), modulation of metallothionein expression (Alvarez et al., 2016), and serves as a cofactor for superoxide dismutase enzyme (Marklund et al., 1982).

The non-enzymatic antioxidants taking part in the first line of defense belong to preventive antioxidants. These antioxidants inhibit the formation of new reactive species by interacting with the transition metal ions (Mironczuk-Chodakowska et al., 2018). Non-enzymatic antioxidants are not only involved in the first line of defense, it also involved in the second line of defense against ROS that is represented by molecules characterized by the ability to inactivate oxidants and radicals (Mironczuk-Chodakowska et al., 2018).

Abundance level of zinc can be found in the retina which implicated the antioxidant defense systems of the eye (Ugarte et al., 2014). Zinc carries out its antioxidant functions and serves as a cofactor in enzymes such as retinol dehydrogenase, an enzyme for vitamin A cycle. Several studies have reported the importance of dietary zinc and age-related macular degeneration. A human study involving 369 participants revealed that a low intake of zinc was associated with age-related macular degeneration (Aoki et al., 2016). A follow-up study for 6 years including 3,640 participants had revealed a significant role of zinc in age-related macular degeneration (Group, 2001). It has been shown that for individuals older than 55 years, zinc supplements may delay the development of age-related macular degeneration and vision loss (Group, 2001). Similarly, a meta-analysis of 23,099 individuals demonstrated that dietary zinc blocks the progression of age-related macular degeneration and delay its progression (Gorusupudi et al., 2017).

In addition to the effects mentioned above, zinc supplementation has been reported to suppress the oxidative stress in type 2 diabetes via several mechanisms. These favorable effects could be attributed to the activity of zinc which is involved in the insulin production, secretion, and action processes by acting as a catalytic cofactor for carboxypeptidase H enzyme. Carboxypeptidase H enzyme is responsible for the conversion of proinsulin (inactive form) into insulin (active form). Further, zinc also facilitates the phosphorylation of the insulin receptor by transporting more glucose into the cells. In human studies, a significant decrease in plasma thiobarbituric acid reactive substances, an oxidative stress indicator was found in patients with type 2 diabetes supplemented with zinc (Anderson et al., 2001). Zinc also improved insulin sensitivity and subsequently reduces the chronic hyperglycemia in type 2 diabetes mellitus (Vashum et al., 2014). Overall, zinc plays a significant role as an antioxidant nutrient that regulates metabolic control in type 2 diabetes mellitus pathophysiology.

Notably, data from epidemiologic studies found that dietary zinc intake may reduce the risk of cancer (Costello and Franklin, 2016). Zinc suppresses the proliferation of cancerous cells via several mechanisms. In cancer cells, zinc inhibits mitochondrial terminal oxidation and respiration and stimulates apoptogenesis of mitochondria (Costello and Franklin, 2016). Further, zinc also prevents the migration of malignant cells through activation of intracellular signaling pathways. In prostate cancer cells, zinc is accumulated in the expression of the zinc uptake transporter, ZIP1 (Franklin and Costello, 2007). The accumulated zinc exerts its antiproliferative activity toward the prostate cancer cells via activation of MAPKs and inhibition the growth of cancer cells (Beyersmann and Haase, 2001). In colorectal cancer, zinc was found to activate Raf-1-MEK-MAPK kinases followed by the activation of Elk-1 dependent trans-reporter gene expression (Park et al., 2002). The downregulation of cancer cell growth by zinc indicates that the therapeutic potential of zinc to regulate the growth of cancers. Taken together, minerals are a good antioxidant which is best supplied by ingesting specific foods rich with that chemical element of interest. The beneficial effect of mineral on aging is worth attention. However, an overdose of mineral intake is not recommended and may cause a detrimental impact on health.

### Ascorbic acid

**Figure 4:**
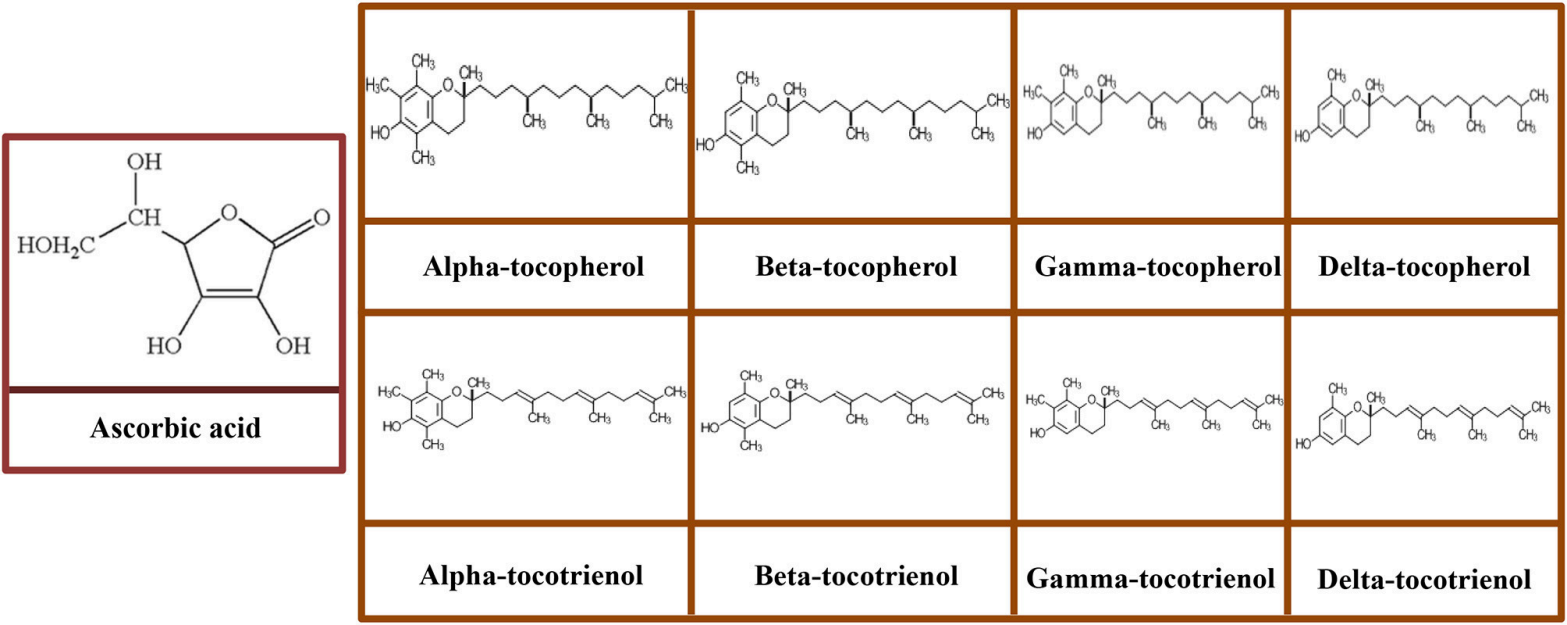
Molecular structures of ascorbic acid and vitamin E congeners including tocopherols (α-tocopherol, β-tocopherol, γ-tocopherol, and δ-tocopherol) and tocotrienols (α-tocotrienol, β-tocotrienol, γ-tocotrienol, and δ-tocotrienol).

Ascorbic acid, also known as vitamin C, is one of the most ubiquitous hydrosoluble antioxidants. In physiological pH conditions, vitamin C exists mainly as an ascorbate anion (Camarena and Wang, 2016). Ascorbic acid has 4 –OH groups (Figure 4) that can donate hydrogen to an oxidizing system. Due to the –OH groups (2 pairs of 2) are on adjacent toward the carbon atoms, ascorbic acid is susceptible to chelate metal ions (Fe^++^). Ascorbic acid serves as a reducing agent, scavenge free radicals, and quench •${\text{O}}_{2}^{-}$. At high levels of ascorbic acid (>1,000 mg/kg), it tends to shift the balance between ferric iron (Fe^3+^) and ferrous (Fe^2+^) and thereby scavenge the oxygen and inhibit oxidation (Brewer, 2011). Ascorbic acid is a cofactor for many enzyme-catalyzed reactions such as maintaining of connective and vascular tissue's integrity, enhancing the collagen biosynthesis and iron absorption, modulating the leukocyte and hematopoiesis functioning, neuroprotection, and hydroxylation of lysine and proline (May and Harrison, 2013; Spector and Johanson, 2014).

Data from both animal and population-based studies have shown that a correlation between the process of aging and reducing ascorbate levels in tissues (Michels and Hagen, 2004; Dixit et al., 2015). The mechanisms that implicate the declining of age-related ascorbate are complex and involve multiple cell signaling pathways such as accelerated turnover, increased usage, reduced cellular uptake, and decreased absorption/reabsorption. For instance, ascorbate level reduces for nearly 50% in leukocytes in individuals at age 85 and above compared to those at age 60 (Attwood et al., 1978). Despite the limited available evidence on ascorbate level in human brains, the previous study has reported that ascorbate level in the cerebral cortex is declined for nearly 77% from an individual at age 80 and older, compared to that individual at age 50 and younger (Schaus, 1957). A study reported by Al-Mahdawi et al. (2014) has shown that ascorbate shortage may contribute to the dysregulation of 5hmC, which subsequently contributes to the age-related neurodegenerative diseases. Research evidence has demonstrated the potential protective function of ascorbate in neurodegenerative diseases (Barnham et al., 2004; Ruszkiewicz and Albrecht, 2015). Ascorbate supplementation markedly improves the differentiation of midbrain derived neural stem cell against dopaminergic neurons, which is associated with the TET-mediated 5hmC generation and Jmjd3 catalyzed loss of H3K27m3 (He et al., 2015). In this regard, these findings imply that ascorbate plays a critical role in dopaminergic neuron differentiation (Camarena and Wang, 2016).

In addition to the effects mentioned above, ascorbic acid has the potential to protect against cancers. High concentration of ascorbic acid induces cytotoxicity against cancer cells *in vitro* (Vuyyuri et al., 2013; Tian et al., 2014) and delays tumor growth in xenograft models (Kim et al., 2012; Ma Y. et al., 2014). The animal model study further demonstrated that feeding *Apc/Kras*^*G*12*D*^ mutant mice high-dose ascorbic acid may impair tumor growth (Yun et al., 2015). Consistent with the data reported by Yun et al. (2015) and Ma Y. et al. (2014), Wu et al. (2017) identify an antitumor activity of ascorbic acid in a clinical study. Although the molecular link underlying ascorbic acid and anticancer activity require further elucidation, most of the experimental studies indicate that modulating oxidative stress could play a crucial role (Badgujar et al., 2015; Huang et al., 2017). In this regard, a key mode of action to explain this relationship is via glucose transporter type 1 (GLUT1) which increases uptake of the oxidized form of ascorbic acid, dehydroascorbate and subsequently depletes glutathione (Yun et al., 2015).

Compared to those who rarely or deficient in ascorbic acid, adults who supplemented with ascorbic acid is negatively associated with adiposity (Hosseini et al., 2017). Several studies have corroborated this finding and found that ascorbic acid suppressed leptin stimulation from adipocytes, particularly in insulin-secreted cells (Garcia-Diaz et al., 2010). Reduction in leptin may trigger a significant reduction in hypertension (Lane and Vesely, 2013). Intriguingly, leptin deficiency is linked to the early-onset of obesity, indicating that ratio of leptin to insulin is fundamental in the homeostatic balance of fat and glucose metabolism (Wabitsch et al., 2015).

Data from a meta-analysis included a study from inception to May 2013 demonstrated that an ascorbic acid supplementation with a dosage > 500 mg/d shows a better endothelial function (Ashor et al., 2014). In a further study focused on cardiovascular disease outcomes, Wang et al. (2013) showed that high ascorbic acid intake is negatively linked to cardiovascular disease. In another meta-analysis of randomized controlled trials, Ashor et al. (2014) found that a relatively low risk for incidence of cardiovascular disease for those with a greater intake of ascorbic acid supplements. Importantly, cellular adhesion molecules are biochemical markers of endothelial dysfunction concomitantly with inflammation. Ascorbic acid was effective by neutralizing the oxidized-low density lipoprotein (oxLDL) activity, which is known as the trigger of the initiator of atherosclerosis and inflammatory process in the endothelial tissue (Ellulu, 2017). Notably, some research has emerged to suggest that ascorbic acid improved endothelial function in diabetic patients (Ashor et al., 2014). The previous study stated that patients with diabetes had relatively low amounts of circulating ascorbic acid concentrations or known as latent scurvy (Price et al., 1996). Taken together, ascorbic acid may be a useful nutritional intervention for the secondary prevention of age-related diseases.

### Vitamin e

Vitamin E consists a group of eight structurally associated lipophilic chromanol congeners. Vitamin E usually found naturally in food including four tocopherols and four tocotrienols, all of which possess saturated and three double bonds in their phytyl tails, respectively. Both tocopherols and tocotrienols are further classified into α-, β-, γ-, and δ- based on the methyl and hydroxyl substitution in their phenolic rings (Figure 4) (Joshi and Pratic, 2012). Among all isoforms of vitamin E, α-tocopherol is predominantly found in mammalian tissue; conversely, γ-tocopherol is the primary form of vitamin E in the diet and exerts potent antioxidant property (Joshi and Pratic, 2012).

The activity of several protein kinases, and particularly of protein kinase C (PKC) sub-family members can be modulated in human neuronal cells supplemented with tocopherols and tocotrienols (Galli et al., 2017). This signaling influences apoptotic cell death and cell cycle regulation in different cell line models such as human neurons and glioblastoma cells with a strong effect of α- and γ-tocopherol on the phosphorylative stimulation of pro-survival MAPK-ERK isoforms. In a close similarity with the results obtained for α-tocotrienols in the post-ischemic brain (Park et al., 2011), α-tocotrienols protect mouse hippocampal and cortical neurons from cell death via modulation of neurodegenerative signaling cascades, and thereby preserve the function of that brain area (Ambrogini et al., 2014). A study by Khanna et al. (2010) and Sen et al. (2007) further supported the role of α-tocotrienol in the modulation of phospholipase A2 activities and 12-lipoxygenase, which are involved in glutamate-induced neuronal cell death.

An emerging role for tocopherols and tocotrienols in response to neuroinflammation has been demonstrated and the occurrence of its positive effects on oxidative damage and Alzheimer pathology has been proposed. The proposed aspects in the neuroinflammatory activity of this vitamin including the regulation of Alzheimer-associated enzymes such as COX-2, 5-lipoxygenase (5-LOX), and nicotinamide adenine dinucleotide phosphate (NADPH) oxidase (Block, 2008; Chu and Praticò, 2011). Further, research evidence indicates that tocopherols and tocotrienols are of benefit in the stimulation of phosphoprotein phosphatase 2A (PP2A), a phosphatase that plays a crucial role in tau homeostasis which is lowered in human Alzheimer's disease brains (Voronkov et al., 2011). Moreover, data from clinical evidence have shown that tocopherols and tocotrienols supplementation in Alzheimer's patients reduces lipid peroxidation by up to 60% compared with that of the control (Morris et al., 2005). In this regard, post-mortem analysis of cerebrospinal fluid found that α-tocopherol levels were positively associated with perceptual speed and Alzheimer's disease pathology in patients (Hensley et al., 2011). Overall, both tocopherols and tocotrienols may induce therapeutic effects via modulation of enzymatic and non-enzymatic pathways to reduce the impairment of neurological function.

Besides its effects on neuroinflammation, previous studies have demonstrated the role of this vitamin as a factor essential for other crucial functions and the development of organs and tissues for example bones, demonstrating the enormous functional potential of tocopherols and tocotrienols. In the context of osteoporosis, tocotrienols therapy has provided significant beneficial outcomes. Gamma-tocotrienol significantly enhanced the secretion levels of osteocalcin and osteonectin, increased alkaline phosphatase activity, and upregulated collagen type I mRNA and Runx2 protein expressions in osteoblastic MC3T3-E1 cells (Xu et al., 2018). Several studies have also reached a similar finding, in which tocopherol and tocotrienol have an antiosteoporotic activity. An animal study has shown that a diet supplemented with γ-tocopherol increased bone mass in male rats (Shuid et al., 2010). Muhammad et al. (2012) and Mohamad et al. (2012) further revealed that feeding with a diet containing α-tocopherol promotes fracture healing and preserves bone mass in the estrogen-deficient rat. Findings from a population-based study mirror some of those from preclinical data obtained from an *in vivo* study. Data from a cross-sectional study showed a positive relationship between bone mineral density and α-tocopherol level in elderly Chinese population (Shi et al., 2016).

In addition to the effects observed on neuroinflammation and osteoporosis, a beneficial effect of tocopherols and tocotrienols supplementation has also been documented on the incidence of cardiovascular disease. The formation of macrophage foam cells is a characteristic and an early onset of atherosclerosis (Yang et al., 2017). The aortas of cholesterol-administered rabbits have typical atherosclerotic lesions and show increased in CD36 mRNA expression. Administration of tocopherols and tocotrienols decreased cholesterol-induced atherosclerotic lesions and downregulated CD36 mRNA expression. The decrease of CD36 scavenger receptor expression, indicating the role of tocopherols and tocotrienols in the reduction of foam cell formation and atherosclerosis (Ozer et al., 2006). These data are in line with the previous study reported *in vitro* for macrophages and human smooth muscle cells, in which α-tocopherol inhibits uptake of oxLDL by downregulating CD36 expression (Devaraj et al., 2001). Previous studies have also reported that feeding Apoe (–/–) mice with tocopherols and tocotrienols downregulated the expression of CD36 and upregulated the transcriptional activity of *LXR*α, *ABCA1*, and peroxisome proliferator-activated receptor gamma (*PPAR*γ) (Tang et al., 2014). A similar dietary supplementation was also found to decrease the phosphorylation of PPARγ and nuclear factor E2-related factor 2 (Nrf2) and induce upregulation of their downstream targets including α-glutathione S-transferase (GSTα) and adenosine triphosphate-binding cassette transporter A1 (ABCA1) through inhibition of matrix metalloproteinase-1 (MMP-1) (Bozaykut et al., 2014). Indeed, data from a meta-analysis included randomized-controlled trials from 1985 to 2015, including 287,304 participants demonstrated that a diet containing tocopherols and tocotrienols is negatively linked to cardiovascular mortality risk (Schwingshackl et al., 2017).

Another age-related disease is arthritis, which causes pain during movement and subsequently promotes loss of function in the affected limb (Espejo-Antúnez et al., 2013). Rossato et al. (2015) reported that tocopherols and tocotrienols reduced pain reversed debilitating symptoms elicited by painful inflammation. The reduction of cytokine production has also been demonstrated in animal models of inflammation and in humans with arthritis (Bhattacharya et al., 2012). Overall, tocopherols and tocotrienols might be promising tools for the alleviation of oxidative stress and preventing age-related diseases. The potential implications of tocopherols and tocotrienols on the age-related diseases worth of further investigation in comparative randomized clinical trials.

### Ubiquinone

**Figure 5:**
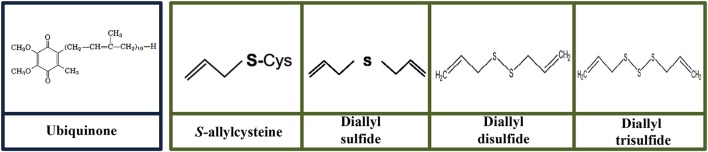
Molecular structures of ubiquinone and organosulfur compounds (*S-*allylcysteine, diallyl sulfide, diallyl disulfide, and diallyl trisulfide).

Ubiquinone (UQ), also known as coenzyme Q10, is synthesized within the body cells or can also be obtained from the diet (Quinzii et al., 2007) (Figure 5). Fish and meat are the richest sources of dietary UQ (Pravst et al., 2010). UQ also can be found in liver, kidney, beef, heart, sardines, soy oil, and peanuts. UQ is a naturally occurring vitamin-like molecule formed from redox-active benzoquinone head group conjugated to a poly-isoprenoid side chain of species-specific length (6–10 subunits) (Wang and Hekimi, 2016). UQ is a potent antioxidant to neutralize ROS and protect the inner lining of the lymph, blood vessels, and endothelium (Motohashi et al., 2017). However, UQ levels reduce with advancing age and subsequently develop to some of the symptoms related to aging. The reduction of UQ levels during aging could be one of the predominant factors to develop chronic diseases (Motohashi et al., 2017).

Compared to other tissue, heart muscle utilizes more energy and usually has the highest UQ level and a relatively sensitive to UQ deficiency. The weakening of the heart muscle may lead to the swelling of the lower legs and feet, lung, liver, and the lining of the intestine (Motohashi et al., 2017). Heart failure is characterized by a loss of contractile function caused by energy depletion in mitochondria linked to a low level of UQ. It was evident that UQ oral supplementation alleviated the endothelial dysfunction and the cardiac contractility (Peres et al., 2017). Congestive heart failure is associated with a low level of UQ in tissues and blood. An animal study showed that UQ reduces the lipid hydroperoxides concentration in atherosclerotic lesions and the atherosclerotic lesions in the aorta (Littarru and Tiano, 2007). Frei et al. (1990) exploring the impact of UQH2 on oxidative stress using liposomes. The data showed that UQH2 protects membrane lipid peroxidation, with similar efficiency as α-tocopherol. oxLDL has been associated with coronary artery disease (Ivanova et al., 2017). Previous study found that the lipid peroxidation rate of human low-density lipoprotein (LDL) is inhibited concomitantly with UQH2 administration following exposure to peroxyl radicals (Stocker et al., 1991). These observations imply that UQ could be one of the most active antioxidants in the modulation of LDL (Wang and Hekimi, 2016). Likewise, data from a population-based study reported that patients with heart failure who consume UQ had a lower risk of mortality in addition to increasing exercise capacity (Lei and Liu, 2017). UQ supplementation may also improve the quality of life in patients with congestive heart failure (Oleck and Ventura, 2016).

In addition to the effects mentioned above, a beneficial role of UQ supplementation has also been observed on the incidence of type 2 diabetes. Data from randomized controlled clinical trials have demonstrated that supplementation with UQ can significantly improve vascular dysfunction and decrease the glycemic response (Mantle, 2017). From the study reviewed, it showed that UQ reduces oxidative stress and did not lead to any adverse effects. Consistent with the study reported by Mantle (2017), Hernández-Ojeda et al. (2012) also found that UQ enhances nerve conduction parameters of diabetic polyneuropathy and ameliorates oxidative stress without significant undesirable effects. A study by Raygan et al. (2016) further supported that UQ increases insulin sensitivity and improves beta cell function in diabetic patients.

UQ plays a central role in the cellular dysfunction of Parkinson's disease patients (Zhu et al., 2017). UQ levels were relatively low in the plasma, platelet-mitochondria, and blood of Parkinson's disease patients (Sohmiya et al., 2004). Treatment with UQ reduced the cellular pathophysiological alterations linked to a mitochondrial dysfunction in Parkinson's disease patients (Cooper et al., 2012). Shults et al. (2002) further demonstrated that high concentration UQ administration may slow down the functional decline experienced by early-stage of Parkinson's disease patients. Overall, lipid profiles, systemic inflammation, and insulin sensitivity were improved after administration of UQ and thus may provide a useful approach for the alleviation of age-related diseases.

To protect mitochondrial oxidative damage, several mitochondrial-targeted antioxidants have been developed and a great potential mitochondrial-targeted antioxidant is MitoQ. MitoQ is a derivative of ubiquinone which linked to TPP moiety by a 10-carbon alkyl chain (Murphy and Smith, 2007). Much information indicates that the ubiquinol moiety of MitoQ can react with superoxide and protect against peroxynitrite (Liu et al., 2018b). Animal studies have revealed that MitoQ protects against oxidative damage such as hypertension (Pak et al., 2018) and neurodegenerative disease (Yin et al., 2016). A study by Junior et al. (2018) further supported that MitoQ improves mitochondrial dysfunction in heart dysfunction induced by pressure overload, by reducing hydrogen peroxide formation in rats. Emerging research evidence indicates that MitoQ may possess beneficial effects on tubular injury (Dare et al., 2015). Notably, data from an animal study have stated that MitoQ inhibited amyloid-β peptide (Aβ) induced oxidative stress, a critical component of Alzheimer's disease, and reversed early cognitive decline (Zhang et al., 2018). Despite the beneficial effects of MitoQ on neurodegenerative disease was reported *in vivo*, not all studies demonstrated such a link. Data from the double-blind clinical trial of Parkinson disease patients failed to show any benefit in delaying the pathologic process of Parkinson disease during 12 months administration of MitoQ (Snow et al., 2010). Overall, these findings demonstrated that MitoQ might be promising tools for pathological changes of mitochondrial oxidative damage associated with age-related diseases.

### Organosulfur compounds

Organosulfur compounds mainly present in vegetable species belonging to *Allium* genus and *Brassicaceae* family. Functional organosulfur compounds in *Allium* have been used in folk and traditional medicine in the last centuries (Petropoulos et al., 2017). Sulfur compounds from *Allium* play a critical role in defense (Nwachukwu et al., 2012). Sulfur is the compound of Fe-S clusters and several amino acids for enzymes activity (Gruhlke and Slusarenko, 2012). Fe-S clusters are vitally important for the origin of life, particularly acetyl-CoA, RNA, and DNA (Fuss et al., 2015). Organosulfur compounds (Figure 5) usually present in onion, garlic, and Chinese chive, which may benefit a certain group of the population because they appear to combat oxidative stress associated age-related diseases such as cardiovascular disease (Lu et al., 2015; Seki and Hosono, 2015; Wang et al., 2015), diabetes (Akash et al., 2014; Sambu et al., 2015), obesity (Lai et al., 2014), and neuroinflammation (Colín-González et al., 2015; Wen and Zhu, 2015). Organosulfur compounds such as *S*-allylcysteine has anti-apoptotic, anti-oxidation, and anti-inflammation (Colín-González et al., 2015) in addition to alleviating several diseases (Kodai et al., 2015).

Several studies reported by Arreola et al. (2015) and Kim et al. (2001) evaluated S-allylcysteine in relation to proinflammatory cytokine production and inflammatory markers. The data showed that S-allylcysteine inhibits NO production and iNOS expression *in vitro* study. Data reported by Liu K. L. et al. (2006) have shown that diallyl disulfide inhibits the production of NO and prostaglandin E2 (PGE2) in lipopolysaccharide (LPS)-stimulated BV2 microglia in a dose-dependent manner. In a further study focused on inflammation outcomes, Liu K. L. et al. (2006) compared different groups of RAW 264.7 cell line murine macrophages that treated with diallyl trisulfide, diallyl disulfide, and diallyl sulfide, respectively. The data demonstrated that diallyl sulfide showed the most suppressive effect on NO production and iNOS expression, suggesting that it was linked to the number of sulfur atoms of the organosulfur compounds.

Organosulfur compounds not only modulate glutathione and phase II enzymes and inhibit inflammatory mediators, they also inhibit several cancers. Previous findings suggest that a diet supplemented with diallyl trisulfide, diallyl disulfide, and diallyl sulfide suppressed cancers induced by chemical carcinogens (Huang J. et al., 2015; Su et al., 2016; Kiesel and Stan, 2017). In support of this, an animal study has demonstrated that oral administration of diallyl trisulfide at a dosage of 1–2 mg per day for 13 weeks significantly suppressed the progression of invasive carcinoma and multiplicity of pulmonary metastasis (Singh et al., 2008). Several studies have also reached a similar finding, in which diallyl trisulfide and diallyl sulfide have an antiproliferative activity against bladder, pancreatic, and skin cancers (Ma H. -B. et al., 2014; Shin et al., 2014; Shan et al., 2016). Collectively, regular consumption of food rich in organosulfur compounds may become a safe and successful strategy to alleviate oxidative stress and improve age-related chronic disease conditions.

Despite antioxidants pharmacological properties were reported *in vitro* and *in vivo*, some of the clinical expectations of antioxidant-based therapies have been frequently disappointed. Low stability and the poor aqueous solubility limit the therapeutic potential of antioxidants. This complication has hampered the quantity of antioxidant absorbed, which severely limit its bioavailability. Nanotechnology has emerged as a promising drug delivery system (Dehghanizade et al., 2018; García Calavia et al., 2018). Nanotechnology has received a great attention as it can resolve problems linked to the conventional therapeutic agents, such as lack of targeting capability, poor water solubility, systemic toxicity, and nonspecific distribution (Sreelakshmi et al., 2018). Hence, the application of nanotechnology could enhance the efficacy and improve their bioavailability by increasing solubility, enhancing plasma half-life, preventing degradation in the intestinal environment, and elevating permeation in the small intestine (Hu et al., 2017).

## Conclusions and future perspective

Despite a large part of literature exploring on the accumulation and the origin of ROS, current antioxidant-based therapies lack of specificity for dysfunctional tissues, cells, and organelles, and thus may not reach an effective concentration at the target site of pathologic oxidative stress. Additionally, antioxidants target huge amounts of reactive oxygen intermediates and are unable to modulate specific intermediate in the oxidative reaction, and subsequently leading several therapeutic strategies are unfocused. Mitochondria-targeted antioxidants hold great promising and may serve as a useful approach for the alleviation of age-related diseases. However, further investigations are warranted to improve the potency of antioxidant-based therapies. Moreover, it is worth to underline the need of exploring the role of iron-mediated oxidative damage through Fenton reaction to further ascertain the contribution of mitochondrial dysfunction and iron accumulation to the progression and pathologic development of age-related diseases. Data available on the interaction between chelating agents and antioxidants are limited, and further investigation may lead to the development of potent therapeutic agents and novel biomarkers targeting specific disease tissues, additionally to identify the downstream mediators of oxidative pathways.

Oxidative stress caused by an overproduction of ROS, mainly due to an imbalance of oxidative to reducing species. It has been suggested that excessive ROS production may lead to an upregulation of oncogene gene and the formation of mutagen compounds, which trigger proatherogenic activity and inflammation. Yet, longevity is not merely embedded in the genes; in fact, food rich in antioxidants may play an essential role in the immune system, production of cellular energy, as well as scavenge the ROS. The broad spectrum of processes in which the antioxidant molecules are involved suggests that a protective role of antioxidants in the pathogenesis of age-related diseases. Thus, an antioxidant can be a useful approach for healthspan extension as well as lifespan extension. Despite antioxidant may not serve as drugs, they hold great promising and indirectly provide leads in future use to combat age-related diseases. The potential implication of antioxidant in relation to age-related diseases to replace conventional therapies could be significant and is warranted to be elucidated in long-term clinical trials.

## Author contributions

BT and W-P-PL conceived and designed the review and wrote the manuscript. MN edited the manuscript. HS wrote the manuscript. All authors read and approved the final manuscript.

### Conflict of interest statement

The authors declare that the research was conducted in the absence of any commercial or financial relationships that could be construed as a potential conflict of interest.

## Abbreviations

ABCA1: adenosine triphosphate-binding cassette transporter A1
AMD: age-related macular degeneration
ATP: adenosine triphosphate
COX: cyclooxygenase
COX-2: cyclooxygenase-2
CRP: C-reactive protein
DDR: DNA damage response
ERK: extracellular signal-regulated kinase
GLUT1: glucose transporter type 1
GSNO: S-nitroglutathione
GSTα: α-glutathione S-transferase
HDL: high-density lipoprotein
HGPS: Hutchinson-Gilford progeria syndrome
HO-1: heme oxygenase-1
H2O2: hydrogen peroxide
ICAM-1: intercellular adhesion molecule 1
IKK: IκB kinase
IL-1β: interleukin-1beta
iNOS: inducible nitric oxide synthase
IL-6: interleukin-6
JNKs: c-Jun N-terminal kinases
LPS: lipopolysaccharide
MAPKs: mitogen-activated protein kinases
MHC: major histocompatibility complex
MMP-1: matrix metalloproteinase-1
NADH: nicotinamide adenine dinucleotide
NADPH: nicotinamide adenine dinucleotide phosphate
NADPH-oxidase: nicotinamide adenine dinucleotide phosphate-oxidase
NCDs: non-communicable diseases
NF-κB: nuclear factor-kappa B
NO: nitric oxide
Nrf-2: nuclear factor E2-related factor 2
O2: oxygen
O2•: superoxide
•OH: hydroxyl radical
ONOO^−^: peroxynitrite
oxLDL: oxidized-low density lipoprotein
PAI-1: plasminogen activator inhibitor-1
PGs: prostaglandins
PGG2: prostaglandin G2
PGH2: prostaglandin H2
PKC: protein kinase C
PPARγ: peroxisome proliferator-activated receptor gamma
PP2A: phosphoprotein phosphatase 2A
PUFAs: polyunsaturated fatty acids
RANKL: receptor activator of nuclear factor kappa B ligand
RNS: reactive nitrogen species
ROS: reactive oxygen species
SAPS: senescence-associated secretory phenotype
TGF-β: transforming growth factor beta
TNF-α: tumor necrosis factor-α
TPP: triphenylphosphonium
Trx: thioredoxin
UQ: ubiquinone
VCAM-1: vascular cell adhesion molecule 1
5-LOX: 5-lipoxygenase.
